# IgG Antibody Responses Are Preferential Compared With IgM for Use as Serological Markers for Detecting Recent Exposure to *Plasmodium vivax* Infection

**DOI:** 10.1093/ofid/ofab228

**Published:** 2021-05-22

**Authors:** Rhea J Longley, Michael T White, Jessica Brewster, Zoe S J Liu, Caitlin Bourke, Eizo Takashima, Matthias Harbers, Wai-Hong Tham, Julie Healer, Chetan E Chitnis, Wuelton Monteiro, Marcus Lacerda, Jetsumon Sattabongkot, Takafumi Tsuboi, Ivo Mueller

**Affiliations:** 1 Population Health and Immunity Division, The Walter and Eliza Hall Institute of Medical Research, Parkville, Australia; 2 Department of Medical Biology, University of Melbourne, Parkville, Australia; 3 Malaria Parasites and Hosts Unit, Department of Parasites and Insect Vectors, Institut Pasteur, Paris, France; 4 Division of Malaria Research, Proteo-Science Center, Ehime University, Matsuyama, Japan; 5 CellFree Sciences Co., Ltd, RIKEN Center for Integrative Medical Sciences, Yokohama, Japan; 6 Infection and Immunity Division, The Walter and Eliza Hall Institute of Medical Research, Parkville, Australia; 7 Malaria Parasite Biology and Vaccines, Department of Parasites and Insect Vectors, Institut Pasteur, Paris, France; 8 Fundacão de Medicina Tropical Dr Heitor Vieira Dourado, Manaus, Brazil; 9 Universidade do Estado do Amazonas, Manaus, Brazil; 10 Instituto Leônicas & Maria Deane (Fiocruz), Manaus, Brazil; 11 Mahidol Vivax Research Unit, Faculty of Tropical Medicine, Mahidol University, Bangkok, Thailand

**Keywords:** antibody, IgG, IgM, malaria, *Plasmodium vivax*, serological exposure markers, serology, surveillance

## Abstract

To achieve malaria elimination, new tools are required to explicitly target *Plasmodium vivax*. Recently, a novel panel of *P. vivax* proteins were identified and validated as serological markers for detecting recent exposure to *P. vivax* within the last 9 months. In order to improve the sensitivity and specificity of these markers, immunoglobulin M (IgM) in addition to immunoglobulin G (IgG) antibody responses were compared with a down-selected panel of 20 *P. vivax* proteins. IgM was tested using archival plasma samples from observational cohort studies conducted in malaria-endemic regions of Thailand and Brazil. IgM responses to these proteins generally had poorer classification performance than IgG.

Infections due to *Plasmodium vivax* are a major challenge for malaria elimination. This is due to unique biological features of *P. vivax* parasites, including an arrested stage in the liver (hypnozoites) that can reactivate weeks to months later, causing relapses of clinical disease. Individuals with hypnozoites are major reservoirs of transmission and are responsible for >80% of blood-stage *P. vivax* infections [1]. Another challenge is the high proportion of low-density, asymptomatic blood-stage infections due to *P. vivax* [2], particularly in low-transmission regions. These factors make it difficult to not only identify infected individuals but also delineate pockets of ongoing local transmission. It is therefore critical that novel tools be developed that enable efficient identification of at-risk individuals who should be targeted for malaria interventions.

We have recently identified and validated a novel panel of *P. vivax* proteins that induce immunoglobulin G (IgG) antibody responses reflective of recent exposure to *P. vivax* blood-stage infections [3]. Combinations of IgG antibody responses to 5–8 *P. vivax* proteins can accurately classify (with 80% sensitivity and specificity) whether an individual has had a *P. vivax* infection within the last 9 months. We chose this 9-month time frame as individuals who have had a detectable blood-stage infection in this period and have not been treated with anti-liver-stage drugs are likely to be harboring hypnozoites in their livers. Our novel serological exposure markers therefore represent the first test that can, indirectly, identify hypnozoite carriers. This tool could play an important role in malaria elimination by offering an alternative to mass drug administration (MDA) strategies (where everyone is treated) or mass screening and treatment (MSAT) strategies performed using currently available diagnostics for blood-stage parasites. While effective, MDA results in a high level of overtreatment. Conversely, MSAT is ineffective using currently available technologies [4]. We have proposed an alternative strategy termed “serological testing and treatment,” whereby individuals are tested using our serological exposure markers and treatment is given to those exposed during the past 9 months.

Here, we investigate the potential utility of alternative biomarkers to IgG antibody responses as serological exposure markers. We previously observed that IgG responses to different *P. vivax* proteins were highly correlated [3], and this is likely why we were unable to improve the classification accuracy by simply incorporating responses to more antigens into the algorithm. Immunoglobulin M (IgM) antibody responses to the same *P. vivax* protein are only weakly correlated to IgG [5] and are generally thought to have a different response kinetic (as shown against *P. falciparum* malaria [6] and other infectious diseases such as West Nile virus [7]). We thus hypothesized that IgM antibody responses to our panel of *P. vivax* proteins could be used to improve the classification accuracy by providing additional information to the algorithm.

## METHODS

We tested our hypothesis using samples from 2 observational cohort studies conducted during 2013–2014: 1 in the Kanchanaburi and Ratchaburi provinces of Western Thailand [8] and 1 in Manaus in the Brazilian Amazon [3]. We utilized plasma samples available from the last visits of these cohorts (n = 829 Thailand, n = 925 Brazil), as previously described [3]. After enrollment, individuals were sampled every month over the yearlong cohort, with 13–14 active case detection visits performed (with polymerase chain reaction [PCR]–based detection of malaria infections). This enabled us to relate IgM (or IgG) antibody levels measured at the last visit with time since previous detected *P. vivax* infection. We also utilized 3 panels of malaria-naïve control plasma samples as previously described [9]: 102 samples from the Volunteer Blood Donor Registry (VBDR), Melbourne, Australia, 100 samples from the Australian Red Cross (ARC), Melbourne, Australia, and 72 samples from the Thai Red Cross (TRC), Bangkok, Thailand.

IgM antibody responses were measured against a panel of 18 or 20 *P. vivax* proteins in samples from the Thai or Brazilian cohorts, respectively (see Supplementary Table 1 for the full list of proteins, expression and purification methods, and sequence regions). These proteins were selected as they performed best when using IgG responses in the first iteration of our algorithm [9]. The *P. vivax* proteins were coupled to nonmagnetic carboxylated microspheres as previously described [10], and IgM levels were measured using a modified multiplexed Luminex assay [3]. Modifications were dilution of plasma samples to 1/200 (instead of 1/100 for IgG) and use of the secondary donkey F(ab’)2 antihuman IgM Fc_5_ (Jackson ImmunoResearch Laboratories, Inc.) at 1/400 dilution. Median fluorescent intensity (MFI) values from the Luminex-200 were converted to relative antibody units based on a standard curve run on each plate generated from a positive control plasma pool consisting of highly immune adults from Papua New Guinea [10]. The same plasma pool was used for the standard curve for both cohorts. Data for KMZ83376.1 and PVX_095055 were not analyzed for the Thai cohort due to a technical issue with the IgM standard curves for the Thai plates. The IgM standard curves had a low starting MFI at the 1/50 dilution of the positive control plasma pool and thus exhibited a plateau in signal from S7-S10, which is not ideal for the standard curve conversion. This issue was subsequently rectified (by generating a new batch of coupled beads), and thus these 2 proteins were assessed in the Brazilian samples. IgG antibody responses against the same *P. vivax* proteins had previously been measured in all samples as described [3].

Individuals from the malaria-endemic cohorts were defined as either (i) infected with *P. vivax* within the 9 months before antibody measurements or (ii) not infected with *P. vivax* within the last 9 months. Individuals from the malaria-naïve control panels were categorized in the latter group. Single-antigen and 2-antigen linear discriminant analysis (LDA) classifications were performed in R studio using R, version 3.5.3 [11], and the packages MASS [12] and ROCR [13]. Single-antigen classification depends on whether a measured antibody level is greater than a defined cutoff. Two-antigen classification depends on the LDA classification score given 2 measured antibody levels.

### Patient Consent

All individuals provided written informed consent or assent, and the studies were approved locally by the Ethics Committee of the Faculty of Tropical Medicine, Mahidol University, Thailand (MUTM 2013-027-01), and the Brazilian National Committee of Ethics (CONEP; 349.211/2013). The Human Research Ethics Committee at WEHI approved the usage of all samples at WEHI and collection of the malaria-naïve control samples (#14/02).

## RESULTS

**Figure 1.**
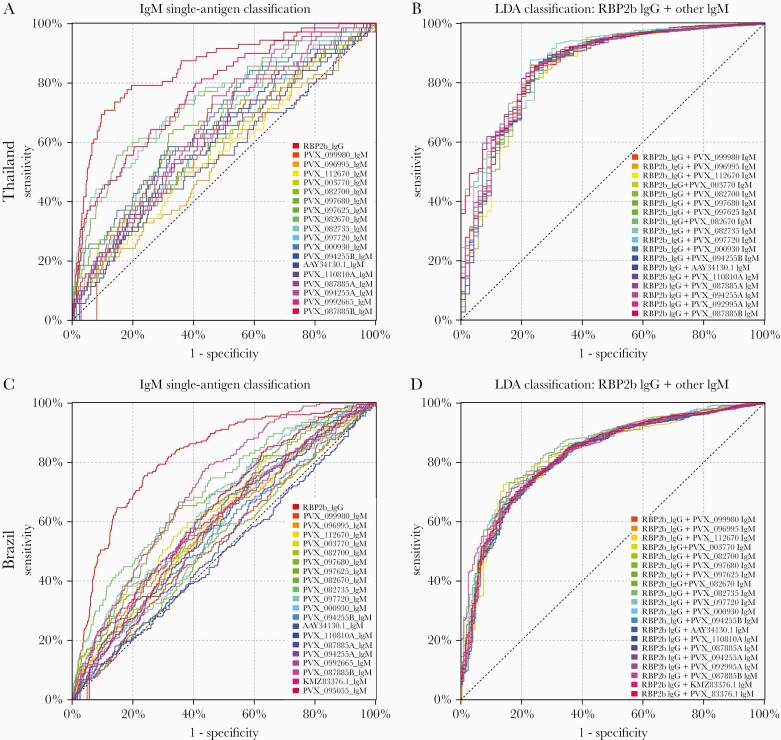
Performance of IgM antibodies against 18–20 *P. vivax* proteins individually (A and C) and in combination with the top-performing protein using IgG (B and D) for classification of *P. vivax* infections in the prior 9 months. In the Thai cohort, IgM antibody responses were measured against 18 *P. vivax* proteins. A, Classification accuracy of these 18 proteins individually, including the top-performing protein for IgG (RBP2b) as reference in red. B, Results from an LDA combining RBP2b IgG with each of the IgM responses to the 18 proteins. In the Brazilian cohort, IgM antibody responses were measured against 20 *P. vivax* proteins. C, Classification accuracy of these 20 proteins individually, including the top-performing protein for IgG (RBP2b) as reference in red. D, Results from an LDA combining RBP2b IgG with each of the IgM responses to the 20 proteins. AUC values are shown in Table 1. Abbreviations: AUC, area under the curve; IgG, immunoglobulin G; IgM, immunoglobulin M; LDA, linear discriminant analysis.

We first determined the accuracy for classifying individuals in the Thai and Brazilian cohorts as recently infected within the last 9 months using IgM antibody responses against 18 or 20 *P. vivax* proteins (Figure 1), respectively. Overall, we observed lower levels of classification accuracy with IgM to these proteins as compared with using IgG [3], as shown in Figure 1 in terms of the top-performing serological exposure marker for IgG (RBP2b). We also measured IgM against the top-performing serological marker RBP2b, which is indicated as PVX_094255B for consistency with the other proteins tested. The area under the curve (AUC) values for IgM ranged from 0.55 to 0.77 for the Thai cohort and 0.50 to 0.72 for the Brazilian cohort (Table 1). In comparison, the AUC values for IgG for the same proteins ranged from 0.70 to 0.85 for the Thai cohort and 0.65 to 0.82 for the Brazilian cohort (Supplementary Table 2) (note that these AUC values are different than that in our prior publication [3] as the current analysis was performed with negative controls from Melbourne and Bangkok only, not including newer samples from Rio de Janeiro). For IgM, the top-performing *P. vivax* protein in both cohorts was PVX_087885B, annotated as the rhoptry-associated membrane antigen (RAMA; putative). One other protein, PVX_082735 (thrombospondin-related anonymous protein [TRAP]), performed well with IgM in both the Thai and Brazilian cohorts, with AUC values of 0.74 and 0.71, respectively. In the Thai cohort, the protein PVX_082670 (merozoite surface protein 7 putative [MSP7]) also performed reasonably well for IgM (AUC, 0.75). The AUCs for IgM responses against RBP2b (the top-performing marker for IgG) were much lower at 0.63 and 0.56 (protein denoted as PVX_094255B in Table 1). IgM antibody responses, stratified by time since previous detected *P. vivax* infection by PCR, are shown in Supplementary Figures 1 and 2.

**Table 1.** AUC Values for Classifying Individuals as Recently Infected With *P. vivax*

	Single-Antigen Classification								LDA	
	1 mo		3 mo		6 mo		9 mo		9 mo	
Protein	Thai	Brazil	Thai	Brazil	Thai	Brazil	Thai	Brazil	Thai	Brazil
RBP2b (IgG)	0.825	0.880	0.856	0.823	0.857	0.823	0.849	0.818	NA	NA
PVX_099980	0.621	0.516	0.601	0.517	0.606	0.529	0.607	0.537	0.855	0.822
PVX_096995	0.551	0.625	0.537	0.591	0.548	0.597	0.549	0.593	0.853	0.822
PVX_112670	0.595	0.590	0.579	0.600	0.585	0.594	0.567	0.589	0.848	0.823
PVX_003770	0.613	0.734	0.573	0.660	0.587	0.644	0.602	0.633	0.852	0.827
PVX_082700	0.665	0.570	0.653	0.613	0.662	0.602	0.641	0.596	0.849	0.828
PVX_097680	0.590	0.551	0.577	0.544	0.593	0.535	0.593	0.529	0.851	0.819
PVX_097625	0.664	0.680	0.647	0.663	0.655	0.655	0.671	0.665	0.856	0.835
PVX_082670	0.786	0.617	0.745	0.650	0.742	0.639	0.747	0.635	0.871	0.827
PVX_082735	0.786	0.774	0.766	0.730	0.758	0.716	0.743	0.705	0.863	0.845
PVX_097720	0.655	0.586	0.629	0.572	0.632	0.568	0.628	0.573	0.858	0.821
PVX_000930	0.709	0.614	0.686	0.634	0.688	0.649	0.673	0.654	0.863	0.835
PVX_094255B	0.628	0.577	0.617	0.545	0.626	0.562	0.631	0.560	0.851	0.819
AAY34130.1	0.622	0.518	0.618	0.519	0.617	0.541	0.597	0.540	0.851	0.819
PVX_110810A	0.580	0.505	0.581	0.475	0.585	0.493	0.554	0.496	0.847	0.818
PVX_087885A	0.628	0.636	0.611	0.602	0.625	0.591	0.615	0.591	0.851	0.823
PVX_094255A	0.673	0.534	0.669	0.555	0.676	0.570	0.675	0.581	0.856	0.821
PVX_092995	0.639	0.584	0.629	0.601	0.644	0.595	0.640	0.599	0.858	0.823
PVX_087885B	0.799	0.733	0.760	0.725	0.771	0.717	0.771	0.715	0.876	0.843
KMZ83376.1	NT	0.623	NT	0.594	NT	0.612	NT	0.617	NT	0.822
PVX_095055	NT	0.616	NT	0.607	NT	0.614	NT	0.624	NT	0.827

LDA (2-antigen combination) was performed using the 9-month classification period with RBP2b IgG plus IgM to 1 of the listed antigens.

Abbreviations: AUC, area under the curve; IgM, immunoglobulin M; LDA, linear discriminant analysis; NA, not applicable; NT, not tested.

As IgM responses are expected to decay more quickly than IgG (due to a shorter serum half-life and their characterization as an early response to infection), we hypothesized that IgM responses may be better markers of very recent exposure. We therefore tested the ability of IgM antibody responses to our 18–20 *P. vivax* proteins to classify individuals as infected with *P. vivax* within the prior 6-, 3-, or 1-month period. As shown in Table 1, for both cohorts, the AUC values did improve when using a shorter time frame for classification. However, the same proteins consistently performed well (PVX_087885, PVX_082735, and PVX_082670) with all classification time frames tested, and the overall improvements were marginal. Furthermore, classification performance with IgM antibodies was still inferior to classification with RBP2b IgG.

Our original results showed that IgG antibody responses to combinations of proteins were better at classifying individuals as infected within the last 9 months compared with individual proteins alone. We therefore tested the classification ability, using the 9-month time frame, of each IgM antibody response combined with IgG responses against the top protein RBP2b. IgM from any of the proteins tested when combined with IgG responses against RBP2b had better classification than IgM alone against any protein, as determined by AUC values (Table 1). In the Thai cohort, all IgM + RBP2b IgG performed (slightly) better than RBP2b IgG alone, with the exception of 2 proteins (PVX_112670 and PVX_110810A). In the Brazilian cohort, all IgM + RBP2b IgG performed (slightly) better than RBP2b IgG alone, with the exception of PVX_110810A. The best combinations were PVX_087885 IgM + RBP2b IgG (AUC, 0.88) for Thailand and PVX_082735 IgM + RBP2b IgG (AUC, 0.85) for Brazil.

## DISCUSSION

Serological markers of recent exposure to *P. vivax* infections could play an important role in malaria elimination by delineating areas of ongoing transmission and identifying individuals with a high chance of carrying hypnozoites in their livers. We note that in our cohort studies the recent blood-stage infections could have been caused by hypnozoites relapsing or by new mosquito-bite derived infections. The fact they have had a recent blood-stage infection means that it is highly likely they are harboring hypnozoites (if they have not received liver-stage drug treatment) and could go on to have future relapses. In this study, we aimed to improve upon an existing set of serological exposure markers by incorporating IgM, in addition to IgG, responses against these proteins. We demonstrate that 2 proteins in particular, PVX_087885 (RAMA) and PVX_082735 (TRAP), induce IgM responses reflective of recent exposure to *P. vivax* within the past 9 months in endemic regions of Thailand and Brazil. They have similar accuracy as compared with these same antigens using IgG (RAMA performs slightly better with IgG; TRAP performs slightly better with IgM). However, the accuracy of these classifications overall is poorer than for the top-performing serological marker (RBP2b) when using IgG, and generally the IgM AUC values for each antigen were lower than the corresponding IgG AUC values (Supplementary Table 2). The poorer performance of IgM responses against these proteins than IgG likely relates to the acquisition and maintenance of IgM antibody responses following *P. vivax* infections. IgM is expected to be short-lived following infection, and supporting this, we find that the classification accuracy does improve if we define recent exposure within a shorter time frame (ie, 1–6 months rather than 9). Another contributing factor is likely the high background of IgM in the non-malaria-exposed controls (Supplementary Figures 1 and 2), compared with our previous results for IgG [3].

When we combined each of the IgM responses against the 18–20 *P. vivax* proteins tested with the IgG response against RBP2b, we demonstrated a clear improvement in classification performance compared with the single-antigen IgM response alone. However, there was only a slight improvement compared with the single-antigen RBP2b IgG alone, signifying that incorporation of IgM responses into the classification algorithm is unlikely to result in substantial improvements in classification. A limitation of our research is that we did not exhaustively test all combinations of IgG and IgM against the down-selected panel of 18–20 *P. vivax* proteins. We also only measured IgM responses against 18–20 of the top *P. vivax* proteins, as indicated by their classification performance when using IgG responses; an alternate approach would have been to measure IgM responses to the full panel of 60 proteins. However, we have already demonstrated that incorporating more than 5 IgG responses results in only marginal improvements in classification performance compared with RBP2b IgG alone, and thus this approach (of exhausting all options) is unlikely to yield better results.

Finally, we ultimately aim to develop a point-of-contact test to be used in the field. It would be a more complicated and costly test if both IgG and IgM responses were required to be measured. We will therefore not be pursuing IgM responses in our optimization of our novel panel of serological exposure markers, and will instead focus on other avenues for improved performance of the signals we can obtain from the IgG responses.

## Supplementary Data

Supplementary materials are available at *Open Forum Infectious Diseases* online. Consisting of data provided by the authors to benefit the reader, the posted materials are not copyedited and are the sole responsibility of the authors, so questions or comments should be addressed to the corresponding author.

ofab228_suppl_Supplementary_Materials

## References

[1] Robinson LJ , Wampfler R, Betuela I, et al. Strategies for understanding and reducing the *Plasmodium vivax* and *Plasmodium ovale* hypnozoite reservoir in Papua New Guinean children: a randomised placebo-controlled trial and mathematical model. PLoS Med 2015; 12:e1001891. 26505753 10.1371/journal.pmed.1001891 PMC4624431

[2] Moreira CM , Abo-Shehada M, Price RN, Drakeley CJ. A systematic review of sub-microscopic *Plasmodium vivax* infection. Malar J 2015; 14:360. 26390924 10.1186/s12936-015-0884-z PMC4578340

[3] Longley RJ , White MT, Takashima E, et al. Development and validation of serological markers for detecting recent *Plasmodium vivax* infection. Nat Med 2020; 26:741–9. 32405064 10.1038/s41591-020-0841-4

[4] Sutanto I , Kosasih A, Elyazar IRF, et al. Negligible impact of mass screening and treatment on mesoendemic malaria transmission at West Timor in Eastern Indonesia: a cluster-randomized trial. Clin Infect Dis 2018; 67:1364–72. 29579195 10.1093/cid/ciy231 PMC6186863

[5] Richards JS , Stanisic DI, Fowkes FJ, et al. Association between naturally acquired antibodies to erythrocyte-binding antigens of *Plasmodium falciparum* and protection from malaria and high-density parasitemia. Clin Infect Dis 2010; 51:e50–60. 20843207 10.1086/656413

[6] Kinyanjui SM , Bull P, Newbold CI, Marsh K. Kinetics of antibody responses to *Plasmodium falciparum*-infected erythrocyte variant surface antigens. J Infect Dis 2003; 187:667–74. 12599084 10.1086/373994

[7] Busch MP , Kleinman SH, Tobler LH, et al. Virus and antibody dynamics in acute west nile virus infection. J Infect Dis 2008; 198:984–93. 18729783 10.1086/591467

[8] Nguitragool W , Karl S, White M, et al. Highly heterogeneous residual malaria risk in Western Thailand. Int J Parasitol 2019; 49:455–62. 30954453 10.1016/j.ijpara.2019.01.004 PMC6996282

[9] Longley RJ , White MT, Takashima E, et al. Development and validation of serological markers for detecting recent exposure to *Plasmodium vivax* infection. bioRxiv 2020; 26:741–9. 10.1038/s41591-020-0841-4 32405064

[10] Longley RJ , França CT, White MT, et al. Asymptomatic *Plasmodium vivax* infections induce robust IgG responses to multiple blood-stage proteins in a low-transmission region of Western Thailand. Malar J 2017; 16:178. 28454546 10.1186/s12936-017-1826-8 PMC5410030

[11] R: A Language and Environment for Statistical Computing. R Foundation for Statistical Computing; 2018.

[12] Venables WN , Ripley BD. Modern applied statistics with S. In: Ripley BD, Venables WN, Masw SP, eds. Statistics and Computing. New York: Springer; 2002.

[13] Sing T , Sander O, Beerenwinkel N, Lengauer T. ROCR: visualizing classifier performance in R. Bioinformatics 2005; 21:3940–1. 16096348 10.1093/bioinformatics/bti623

