# Supplementary material for: IgG Antibody Responses Are Preferential Compared With IgM for Use as Serological Markers for Detecting Recent Exposure to Plasmodium vivax Infection
Source: Open Forum Infect Dis. 2021 May 22;8(6):ofab228. doi: 10.1093/ofid/ofab228 (PMC8214013; doi:10.1093/ofid/ofab228)
Supplement: ofab228_suppl_Supplementary_Materials [file ofab228_suppl_supplementary_materials.docx]

**Supplementary Information**

Figure S1: Measured IgM antibody response to 18 *P. vivax* proteins on the Luminex® platform, stratified by geographical location (Thailand or controls) and time since last PCR detected infection. RAU = relative antibody units, calculated against the standard curve included on each plate.

Figure S2: Measured IgM antibody response to 20 *P. vivax* proteins on the Luminex® platform, stratified by geographical location (Brazil or controls) and time since last PCR detected infection. RAU = relative antibody units, calculated against the standard curve included on each plate.

Table S1: *P. vivax* proteins used in this study. Proteins were selected based on our previously validation study as being able to accurately classify individuals as infected with *P. vivax* in the last 9-months when using IgG antibody responses, either individually or in combination [1]. References listed are for the protein production and purification method. Protein ID and annotations are from PlasmoDB (release 36 <http://plasmodb.org/plasmo/>) or GenBank. Where the protein ID has an A or B, it is used to match exactly to the proteins listed in our validation manuscript. The Short Code listed is in reference to our original manuscript [1].

| Protein ID | Short Code | Gene Annotation | Protein length, aa | Construct, aa (size) | Expression System | Purification Method |
| --- | --- | --- | --- | --- | --- | --- |
| PVX_099980 | W01 | merozoite surface protein 1 (MSP1-19) | 1751 | 1622-1729 (108) | WGCF | One-step Ni column |
| PVX_096995 | W02 | tryptophan-rich antigen (Pv-fam-a) | 480 | 61-end (420) | WGCF | One-step Ni column |
| PVX_112670 | W08 | unspecified product | 335 | 34-end (302) | WGCF | One-step Ni column |
| PVX_003770 | W12 | merozoite surface protein 5 (MSP5) | 387 | 23-365 (343) | WGCF | One-step Ni column |
| PVX_082700 | W27 | merozoite surface protein 7 (MSP7.1) | 420 | 23-end (397) | WGCF | One-step Ni column |
| PVX_097680 | W28 | merozoite surface protein 3 (MSP3.3) | 1016 | 21-end (996) | WGCF | One-step Ni column |
| PVX_097625 | W30 | merozoite surface protein 8 (MSP8), putative | 487 | 24-463 (440) | WGCF | One-step Ni column |
| PVX_082670 | W31 | merozoite surface protein 7 (MSP7), putative | 411 | 24-end (388) | WGCF | One-step Ni column |
| PVX_082735 | W34 | thrombospondin-related anonymous protein (TRAP) | 556 | 26-493 (468) | WGCF | One-step Ni column |
| PVX_097720 | W39 | merozoite surface protein 3 (MSP3.10) | 852 | 25-end (828) | WGCF | One-step Ni column |
| PVX_000930 | W40 | sexual stage antigen s16, putative | 140 | 31-end (110) | WGCF | One-step Ni column |
| PVX_094255B | W50 | reticulocyte binding protein 2b (RBP2b) | 2806 | 161-1454 (1294) | *E. coli* | 2x affinity + size exclusion [2] |
| AAY34130.1 | W57 | Duffy binding protein (DBP, region 2, AH strain) | 237 | 1-237 (237) | *E. coli* | Ni, ion exchange, gel filtration [3] |
| PVX_110810A | W53 | Duffy binding protein (DBP, region 2, Sal1 strain) | 1070 | 193-521 (329) | *E. coli* | Ni, ion exchange, gel filtration [3, 4] |
| PVX_087885A | W16 | rhoptry associated membrane antigen, putative | 730 | 462-730 (269) | WGCF | One-step Ni column |
| PVX_094255A | W20 | reticulocyte binding protein 2b (RBP2b) | 2806 | 1986-2653 (667) | WGCF | One-step Ni column |
| PVX_092995 | W46 | tryptophan-rich antigen (Pv-fam-a) | 385 | 25-358 (334) | WGCF | One-step Ni column [5] |
| PVX_087885B | W47 | rhoptry-associated membrane antigen, putative | 730 | 462-730 (269) | WGCF | One-step Ni column [5] |
| KMZ83376.1 | W58 | erythrocyte binding protein II (PvEBPII) | 786 | 109-432 (324) | *E. coli* | Ni, ion exchange, gel filtration [3, 4] |
| PVX_095055 | W55 | Rh5 interacting protein, putative (RIPR) | 1075 | 552-1075 (524) | *E. coli* | 2x affinity + size exclusion [6] |

Table S2: Direct comparison of AUC values for each protein for IgG versus IgM, for classifying recent infection in the past 9 months.

|  | Thailand | | Brazil | |
| --- | --- | --- | --- | --- |
| Antigen | IgM | IgG | IgM | IgG |
| PVX_099980 | 0.607 | 0.8117523 | 0.537 | 0.7870332 |
| PVX_096995 | 0.549 | 0.7457229 | 0.593 | 0.7243792 |
| PVX_112670 | 0.567 | 0.7553279 | 0.589 | 0.7306483 |
| PVX_003770 | 0.602 | 0.7530849 | 0.633 | 0.6703616 |
| PVX_082700 | 0.641 | 0.7587684 | 0.596 | 0.7436841 |
| PVX_097680 | 0.593 | 0.7049692 | 0.529 | 0.6507685 |
| PVX_097625 | 0.671 | 0.7670301 | 0.665 | 0.7456572 |
| PVX_082670 | 0.747 | 0.7268631 | 0.635 | 0.6978052 |
| PVX_082735 | 0.743 | 0.7019698 | 0.705 | 0.6598704 |
| PVX_097720 | 0.628 | 0.7894644 | 0.573 | 0.7270199 |
| PVX_000930 | 0.673 | 0.7899103 | 0.654 | 0.7811927 |
| PVX_094255B | 0.631 | 0.849134 | 0.560 | 0.8177998 |
| AAY34130.1 | 0.597 | 0.7397995 | 0.540 | 0.772517 |
| PVX_110810A | 0.554 | 0.7155953 | 0.496 | 0.7613065 |
| PVX_087885A | 0.615 | 0.7588708 | 0.591 | 0.7527885 |
| PVX_094255A | 0.675 | 0.8051379 | 0.581 | 0.7624446 |
| PVX_092995 | 0.640 | 0.7917883 | 0.599 | 0.7028894 |
| PVX_087885B | 0.771 | 0.8065824 | 0.715 | 0.7481328 |
| KMZ83376.1 | N/A | N/A | 0.617 | 0.7393093 |
| PVX_095055 | N/A | N/A | 0.624 | 0.7717238 |

**References:**

1. Longley RJ, White MT, Takashima E, et al. Development and validation of serological markers for detecting recent exposure to <em>Plasmodium vivax</em> infection. bioRxiv **2018**:481168.

2. Hietanen J, Chim-Ong A, Chiramanewong T, et al. Gene Models, Expression Repertoire, and Immune Response of Plasmodium vivax Reticulocyte Binding Proteins. Infect Immun **2015**; 84:677-85.

3. Cole-Tobian JL, Michon P, Biasor M, et al. Strain-specific duffy binding protein antibodies correlate with protection against infection with homologous compared to heterologous plasmodium vivax strains in Papua New Guinean children. Infect Immun **2009**; 77:4009-17.

4. Franca CT, White MT, He WQ, et al. Identification of highly-protective combinations of Plasmodium vivax recombinant proteins for vaccine development. Elife **2017**; 6.

5. Lu F, Li J, Wang B, et al. Profiling the humoral immune responses to Plasmodium vivax infection and identification of candidate immunogenic rhoptry-associated membrane antigen (RAMA). J Proteomics **2014**; 102:66-82.

6. Healer J, Thompson JK, Riglar DT, et al. Vaccination with conserved regions of erythrocyte-binding antigens induces neutralizing antibodies against multiple strains of Plasmodium falciparum. PLoS One **2013**; 8:e72504.
